# The Need for Registries in the Early Scientific Evaluation of Surgical Innovations

**DOI:** 10.3389/fsurg.2014.00012

**Published:** 2014-04-28

**Authors:** Ferdinand Köckerling

**Affiliations:** ^1^Department of Surgery and Center of Minimally Invasive Surgery, Vivantes Hospital, Academic Teaching Hospital of Charité Medical School, Berlin, Germany

**Keywords:** registry, randomized controlled trials, surgical innovation, prospective research database, NOTES registry

As a field, surgery has generated some of the leading innovators in history (1). Advances in immunology and microbiology knowledge and exciting technological developments, e.g., 3D imaging, robotic surgery, natural orifice transluminal endoscopic surgery, tissue engineering, and 3D printing, will maintain the innovation potential in the field of surgery on a high level.

Randomized controlled trials (RCTs) represent the gold standard for evaluation of the safety and efficacy of surgical interventions. There are several factors that make conduct of RCTs of surgical procedures particularly difficult. Therefore in the past the majority of surgical innovations were accepted on the basis of non-randomized trials (2).

That was highlighted, in particular, by the reaction to the introduction of laparoscopic cholecystectomy, which was greeted by Sir Alfred Cushieri as the “greatest unaudited procedure in the history of surgery” (3).

The first minimally invasive gallbladder-removal procedure was performed by Mühe in Germany in 1985 using a galloscope he himself had designed (4), and the first video-endoscopic cholecystectomy was carried out by Mouret in France in 1987 (3). The first case series was then published by Dubois in 1989 with 63 cases (5), and by Perissat in 1992 with 777 cases (6). By that time, laparoscopic cholecystectomy had already become established in many hospitals worldwide. A problematic issue during that phase of “scientific uncertainty” was the significantly higher rate of common bile duct injuries, especially during the learning curve (7).

The first report on a prospective randomized trial comparing laparoscopic with open cholecystectomy were published in 1992, attesting to the benefits of the minimally invasive technique (8). However, the sample size in that first RCT was only 70 patients. It was only in 2006, when the Cochrane Collaboration reviewed in a meta-analysis of 38 RCTs with 2,338 patients, i.e., on average 62 patients per study, that it was possible to issue a scientifically corroborated statement demonstrating that laparoscopic cholecystectomy did not differ from the open technique in terms of mortality, complication rate, or operating time, but did result in a shorter hospital stay and quicker convalescence (9).

The authors thus concluded that “these results confirm the existing preference for the laparoscopic cholecystectomy over open cholecystectomy.”

It thus took 20 years from the initial introduction of video-endoscopic cholecystectomy until scientific proof of its benefits to the patients could be demonstrated. That proof, of the highest level of evidence according to the Oxford criteria, was obtained from the RCT gold standard and meta-analysis. By that time, laparoscopic cholecystectomy had already become established as the gold standard in all hospitals worldwide.

As a reaction to the analysis of how innovations were taking place in surgery, an expert group was set up within the framework of the Balliol Collaboration to compile recommendations for scientific evaluation of surgical innovations. In that spirit, McCulloch (10) stated that “in the short term, we cannot change how surgical innovations happen and so we need to adapt our methods to the process rather than doing the opposite.”

In addition, the Balliol Collaboration highlighted the special features of this surgical innovation process: “By contrast with the formalized approach for drug developments, the process in surgery has been unregulated, unstructured, and variable. Surgery and other invasive therapies are complex interventions, the assessment of which is challenged by factors that depend on operator, team, and setting, such as learning curves, quality variations, and perception of equipoise” (10).

To take account of how surgical innovations take place in reality, the Balliol Collaboration recommends that details of patients treated with the new technique be recorded in prospective development studies, prospective research databases, or prospective registries. These prospectively recorded data will provide for a better power calculation for RCTs, indications can be formulated for this new technique and quality criteria identified (10). As such, prospectively recorded registry data can play an important role in the development of high-caliber RCTs since the prospective registry data can pave the way for an enhanced study design for RCTs. In particular, this would cut back on the need for RCTs with a relatively small sample size, while reserving resources for those RCTs endowed with adequate power.

A good example of early scientific evaluation of surgical innovation by means of a prospective research database is the NOTES registry of the German Society of General and Visceral Surgery. Data on all procedures related to natural orifices (transgastral, transrectal, and transvaginal) can be entered into the registry for Natural Orifice Transluminal Endoscopic Surgery. The national NOTES registry was set up by the German Society of General and Visceral Surgery to collect data on implementation of the new technique. The aim is to draw on past experiences gathered at the time of introducing minimally invasive surgery and identify any problems at an early stage. By the end of February 2014, data on 3,211 NOTES operations had been entered into the registry. The initial results of 551 procedures were published in 2010 in *Annals of Surgery* (11). These data demonstrated that transvaginal cholecystectomy can be performed to the required safety standard as an alternative to laparoscopic cholecystectomy.

Another example is the various hernia registries (Herniamed, Danish Hernia Registry, Swedish Hernia Registry, EuraHS, etc.). Hernia surgery, in particular, has experienced rapid progress in recent years. By virtue of the ever-expanding number of medical devices used in hernia surgery (meshes, tackers, and sealants), the surgical techniques are of such a broad variety that they can scarcely be evaluated in a RCT. But by consistently recording details of the different surgical techniques in a prospective registry, any problems or complications related to particular variants of the technique can be identified at an early stage (12).

The voluntary recall of a metal-on-metal hip implant from the market around the world is an excellent example for the validity of registries in the early identification of complications with a specific surgical procedure. The reason that the company has done this was due to the analysis of data from the Australian National Joint Replacement Registry demonstrating that the rate of early revision surgery for these implants was higher relative to other hip replacements. Additionally, there are concerns about metal-on-metal hip implants and the possibility of adverse health consequences of metal particles and metal ions that may generated by wear of the components of these implants (13).

In summary, the innovation process in surgery is essentially more unstructured, unregulated, and variable than in conservative medicine. Scientific evaluation of surgical innovations must be adapted to take account of that. In addition to the gold standard of evidence-based medicine, i.e., the RCT, surgical innovations should in parallel involve prospective recording of data on patients in a database, i.e., a registry. This will provide for early identification of any problems or complications on the basis of outcome analysis. In the surgical innovation process, registries constitute an important scientific tool that affords insights from the outset and accordingly merits evaluation. All scientists engaged in surgical innovations are called upon to support and promote the development of such registries. Surgeons who themselves create innovations should enter data into a registry on patients treated as per the innovative technique. The challenge is getting surgeons to be more critical about the outcomes of their innovations.

## Conflict of Interest Statement

The author declares that the research was conducted in the absence of any commercial or financial relationships that could be construed as a potential conflict of interest.
